# Real-world treatment patterns in stage III and IV melanoma patients: insights from a global multi-center chart review study

**DOI:** 10.3389/fimmu.2026.1813387

**Published:** 2026-07-23

**Authors:** Miso Kim, Elizabeth M. Gaughan, Agueda Azpeitia, Aparna Rao, Maria L. Wei, Alexandra So, Xiaochen Zhong, Carola Berking, Ruixuan Jiang, Stéphane Dalle, Caroline Robert, Salma Alam, Tessa Davies, Dirk Debus, Marcin Dzienis, Sarah Danson, Ricky Frazer, Christoffer Gebhardt, Glenn Geidel, Jessica C. Hassel, Inga Hansen, Markus V. Heppt, Lina Hildebrandt, James Isaacs, Koung Jin Suh, Bhumsuk Keam, Yu Jung Kim, Thierry Lesimple, Phillipe Saiag, Ryan Weight, Alicia Delibes, Rosemarie Barnett, Clemens Krepler, Kavita Gandhi, Nawab Qizilbash, Irene Shui, Xiang-Lin Tan, Ryan J. Sullivan

**Affiliations:** 1 Seoul National University Hospital, Seoul, Republic of Korea; 2 University of Virginia Cancer Center, Charlottesville, VA, United States; 3 OXON Epidemiology, Madrid, Spain; 4 Department of Medical Oncology, Peter MacCallum Cancer Centre, Melbourne, VIC, Australia; 5 Melanoma Research Victoria, Melbourne, Australia; 6 University of California, San Francisco, San Francisco, CA, United States; 7 San Francisco VA Health Care System, San Francisco, CA, United States; 8 Department of Dermatology, Deutsches Zentrum Immuntherapie, Universitätsklinikum Erlangen, CCC Erlangen-EMN, Friedrich-Alexander-Universität Erlangen-Nürnberg, Erlangen, Germany; 9 Merck Sharp & Dohme LLC (MSD), a subsidiary of Merck & Co., Inc., Rahway, NJ, United States; 10 Centre Hospitalier Lyon-Sud, Pierre-Benite Cedex, France; 11 Institut Gustave Roussy, Villejuif, France; 12 Mount Vernon Cancer Centre, Northwood, United Kingdom; 13 Velindre University NHS Trust, Cardiff, United Kingdom; 14 Department of Dermatology, Nuremberg Hospital, Paracelsus Medical University, Nuremberg, Germany; 15 Gold Coast University Hospital, Southport, QLD, Australia; 16 Weston Park Cancer Centre, University of Sheffield and Sheffield Teaching Hospitals NHS Foundation Trust, Sheffield, United Kingdom; 17 Department of Dermatology and Venereology, University Medical Center Hamburg-Eppendorf, Hamburg, Germany; 18 Heidelberg University, Medical Faculty Heidelberg, Department of Dermatology and National Center for Tumor Diseases (NCT), NCT Heidelberg, a partnership between DKFZ and University Hospital Heidelberg, Heidelberg, Germany; 19 Dermpath München, Laboratory for Dermatopathology, Oral Pathology and Molecular Pathology, Munich, Germany; 20 Cleveland Clinic, Cleveland, OH, United States; 21 Seoul National University Bundang Hospital, Seoul National University College of Medicine, Seongnam, Republic of Korea; 22 Centre Eugéne Marquis, Rennes, France; 23 Department of General and Oncologic Dermatology, Ambroise Paré hospital, APHP, & EA 4340 “Biomarkers in cancerology and hemato-oncology”, UVSQ, Université Paris-Saclay, Boulogne-Billancourt, France; 24 The Melanoma and Skin Cancer Institute, Englewood, CO, United States; 25 University of Bath, Bath, United Kingdom; 26 Royal National Hospital for Rheumatic Diseases, Bath, United Kingdom; 27 The London School of Hygiene & Tropical Medicine, London, United Kingdom; 28 Mass General Brigham Cancer Institute, Boston, MA, United States

**Keywords:** adjuvant setting, advanced, anti-PD-1, melanoma, real-world data, treatment patterns

## Abstract

**Introduction:**

Anti-PD-1 immune checkpoint inhibitors have substantially changed melanoma management in both the adjuvant and advanced settings. This study aimed to estimate the percentage of stage III and IV melanoma patients exposed to different therapies and describe treatment patterns among anti-PD-1-treated patients.

**Methods:**

We performed a retrospective chart review across 22 centers in Australia, France, Germany, South Korea, the United Kingdom, and the United States. Two patient populations were analyzed: (1) an aggregate cohort with stage III/IV melanoma (2018-2021), and (2) patients who received anti-PD-1 therapy in adjuvant or advanced settings from January 2018–12 months before data collection. Treatment patterns were evaluated.

**Results:**

In the aggregate cohort (N = 16,260), observation/watchful waiting predominated in stage III patients (56.8%) in the adjuvant setting, whereas systemic therapy was most common for stage IV disease in the advanced setting. In the anti-PD-1 cohort (N = 738), anti-PD-1 monotherapy predominated in the adjuvant setting (97.5%), while both anti-PD-1 monotherapy (63.1%) and anti-PD-1 plus anti-CTLA-4 (36.9%) were commonly used in the advanced setting. Across successive lines of therapy, anti-PD-1-based regimens remained frequently used despite substantial attrition between treatment lines. Disease progression was the leading cause of treatment discontinuation in advanced disease.

**Discussion:**

This real-world study highlights the central role of anti-PD-1 therapy across disease stages. Anti-PD-1 retreatment and reuse of anti-PD-1-based regimens were frequently observed across treatment lines, highlighting the limited availability of established post-anti-PD-1 treatment options. Novel strategies are urgently needed for patients progressing after anti-PD-1 therapy.

## 1 Introduction

The introduction of immune checkpoint inhibitors (ICIs) has reshaped the therapeutic landscape for melanoma across both the adjuvant and advanced settings. In advanced disease, ipilimumab was the first ICI to demonstrate durable benefit, leading to United States (US) Food and Drug Administration (FDA) and European Medicines Agency (EMA) approval in 2011 (1). The subsequent approval of the anti-PD-1 agents nivolumab and pembrolizumab for unresectable or advanced melanoma in the US and European Union in 2014–2015 further improved outcomes (2–5). Combination nivolumab plus ipilimumab was approved by the FDA for advanced disease in 2015, after demonstrating improved response rates and progression-free survival with durable efficacy, albeit with increased toxicity relative to either agent alone (6). Most recently, combination nivolumab plus relatlimab (anti-LAG-3) was approved for advanced melanoma in 2022 following the RELATIVITY-047 trial, which showed superior progression-free survival versus nivolumab monotherapy, and a more favorable toxicity profile than other immunotherapy combinations (1, 7, 8). In the adjuvant setting, nivolumab (approved in 2017), significantly improved recurrence-free survival compared with ipilimumab in resected stage III-IV melanoma in the CheckMate-238 trial (9). Pembrolizumab was subsequently approved by the EMA (2018) and FDA (2019) for adjuvant treatment of resected stage III melanoma based on results from the KEYNOTE-054 (EORTC 1325) trial, which demonstrated sustained improvements to recurrence-free and distant metastasis-free survival versus placebo (10–13).

Earlier real-world studies reflected a therapeutic landscape before the widespread adoption of ICIs. A multi-country European study in patients with unresectable stage III- IV melanoma reported the predominant use of dacarbazine, fotemustine, or temozolomide, with poor survival outcomes (14). With the advent of targeted therapies, treatment decisions were increasingly shaped by BRAF mutational status, as shown by a French retrospective chart review of patients with unresectable stage III/IV melanoma, where most BRAF-mutant patients received BRAF inhibitors, while wild-type patients continued to receive chemotherapy (15). More recent real-world studies have begun documenting shifts following ICI introduction. A US study reported that ipilimumab and nivolumab monotherapy were the most common first-line of therapies (LOT) in 2015 and 2016, respectively (16). Similarly, an analysis of the Flatiron Health database (2014–2016) documented a rapid uptake of anti-PD-1 agents in both BRAF-mutant and wild-type patients following the FDA approval of anti-PD-1 agents in late 2014, with a marked decline in ipilimumab-based regimens and BRAF inhibitor monotherapy (17).

Collectively, available evidence illustrates a substantial evolution in melanoma treatment paradigms over the past two decades. However, contemporary real-world evidence describing treatment selection, sequencing, and management in the ICI era remains limited, particularly following progression on anti-PD-1 in the adjuvant setting. Improved understanding of real-world management in this population is critical to identify unmet medical needs, inform clinical decision-making and sequencing strategies, and guide future prospective research. To address these knowledge gaps, we conducted a multi-country, retrospective chart review to characterize contemporary real-world treatment utilization and sequencing patterns in stage III/IV melanoma, with particular focus on management following initiation of anti-PD-1 therapy in the adjuvant and advanced settings.

## 2 Methods

### 2.1 Study design and study population

This non-interventional chart-review study was conducted in medical oncology or dermato-oncology departments at 22 sites in Australia, France, Germany, South Korea, the United Kingdom, and the United States. Two study cohorts were defined. An aggregate cohort included all patients aged ≥18 years with histologically confirmed stage III or IV melanoma who received treatments as part of routine clinical practice between 01 January 2018 and 31 December 2021. The purpose of the aggregate cohort was to provide a snapshot of the proportion of patients who received anti-PD-1-based therapy, BRAF-targeted therapy, and other treatments using cumulative site-level data.

We then conducted a more detailed chart review to obtain more granular longitudinal data on treatment patterns, treatment sequencing, persistence, and management following initiation of anti-PD-1 therapy for a cohort of patients who received anti-PD-1-based therapy (anti-PD-1 cohort).

The anti-PD-1 cohort comprised patients aged ≥18 years with histologically confirmed stage III or IV cutaneous melanoma who initiated anti-PD-1 therapy in either the adjuvant (following complete resection of stage III or IV disease) or advanced (unresectable or metastatic disease) setting between 1 January 2018 and a cutoff of 12 months before start date of each site’s data collection. Eligible patients were required to have received at least two cycles of anti-PD-1 therapy and to have a minimum follow-up of 12 months, unless death occurred earlier. Exclusion criteria were non-cutaneous melanoma subtypes (e.g., uveal or mucosal), receipt of fewer than two cycles of anti-PD-1 therapy, enrollment in an interventional clinical trial, or the presence of another active malignancy at anti-PD-1 initiation. For the detailed chart review of the anti-PD-1 cohort, we pre-specified a target enrolment of approximately 750 patients, which were distributed among 22 sites based on country and sample size. Within each site and treatment setting (adjuvant and advanced), patients were screened and enrolled consecutively from 01 January 2018 until predefined site-level and global enrollment targets were reached. This sampling approach was intended to capture a broad, multi-country sample of anti-PD-1-treated patients and ensure sufficient numbers of patients in both the adjuvant and advanced settings for descriptive analyses. The chart review cohort was therefore not designed to be a proportional random sample of all anti-PD-1-treated patients in the aggregate cohort.

The study was conducted in accordance with local regulatory and ethical requirements.

### 2.2 Data collection and outcomes

For the aggregate cohort, cumulative site-level data from 20 of the 22 participating sites were collected between July 2022 and February 2024. Data were recorded using a standardized collection form that captured, for each site, the numbers of patients with stage IIIA, IIIB, IIIC, IIID, and IV melanoma, stratified by age group, gender, and type of treatment in the adjuvant and advanced settings over the study period. The primary outcomes were the proportion of patients with stage III or IV melanoma who received anti-PD-1-based therapy, BRAF-targeted therapy, or other systemic treatments between 1 January 2018 and 31 December 2021.

For the anti-PD-1 cohort, more detailed clinical data, including baseline demographic, disease history, and clinical characteristics, were collected between March 2023 and May 2024 via retrospective chart review. The index date was defined as the initiation of first-line anti-PD-1-based therapy (i.e., index LOT). The end of the index LOT was determined by the permanent discontinuation of the regime, an addition, or a switch. The index LOT was characterized by regimen type, duration of anti-PD-1 treatment, temporary interruptions, permanent interruptions, concomitant medications, permanent discontinuation, persistence, and reasons for discontinuation. Temporary interruptions were defined as cessation of one or more treatment components for less than 120 days and were not considered a change in LOT. Permanent interruptions were defined as lasting modifications to a treatment regimen (i.e., switching agents; removal of an agent, addition of a new agent, LOT discontinuation, or oncologic treatment discontinuation) and were considered as LOT modifications. Persistence was defined as the proportion of patients remaining on their index anti-PD-1 therapy without permanent discontinuation at given time intervals.

Overall, anti-melanoma therapy, including surgery, radiotherapy, and systemic therapies (other than index LOT) was assessed at three timepoints: (1) before index date, (2) after index date but before disease progression (defined as per physician criteria), and (3) after disease progression, stratified by stage and treatment setting. Characteristics of the index LOT were further analyzed separately for patients with and without disease progression in both settings. The time to permanent discontinuation of the index LOT was assessed. Successive LOTs were evaluated up to the fourth LOT and followed through to the end of the study period. Outcomes for each LOT included regimen composition, duration, participation in a clinical trial, and use of concomitant medications. Analyses for each LOT were restricted to patients who proceeded to the respective subsequent LOT; therefore, denominators differed across LOTs because of attrition over time.

### 2.3 Statistical analysis

All statistical analyses were performed using SAS software, version 9.4 (2025; SAS Institute Inc., Cary, NC, USA). We focused on the descriptive analyses prespecified in the statistical analysis plan, including characterization of treatment utilization, sequencing patterns, treatment duration, and discontinuation across adjuvant and advanced settings. Descriptive analyses were presented separately by disease setting/stage, successive LOT, and country where applicable.

For the aggregate cohort, the proportion of melanoma patients who received treatments was estimated using the cumulative site-level dataset. Frequencies and percentages were reported and corresponding 95% confidence intervals (CI) were calculated for patients who received anti-PD1-based therapy, BRAF-targeted therapy, other systemic treatments, or observation/watchful waiting, stratified by disease stage and treatment setting.

For the anti-PD-1 cohort, the planned sample size was 750 patients, which was calculated to estimate a 10% proportion with a margin of error of ±2.15% at a 95% CI. Categorical variables were summarized by frequency and percentage, and continuous variables were summarized by mean and standard deviation (SD). Treatment duration was presented as median with interquartile range (Q1-Q3) and persistence at prespecified time points was estimated by Kaplan-Meier analysis, with corresponding Clopper-Pearson 95% CIs. Univariate comparisons of the index LOT characteristics between patients with and without progression were performed using Pearson’s chi-square test for categorical variables, and either t-test or Mann-Whitney’s U for continuous variables, depending on their normality. Treatment pathways were described using Sankey diagrams.

## 3 Results

### 3.1 Patient characteristics and treatments in the aggregate cohort

**Figure 1:**
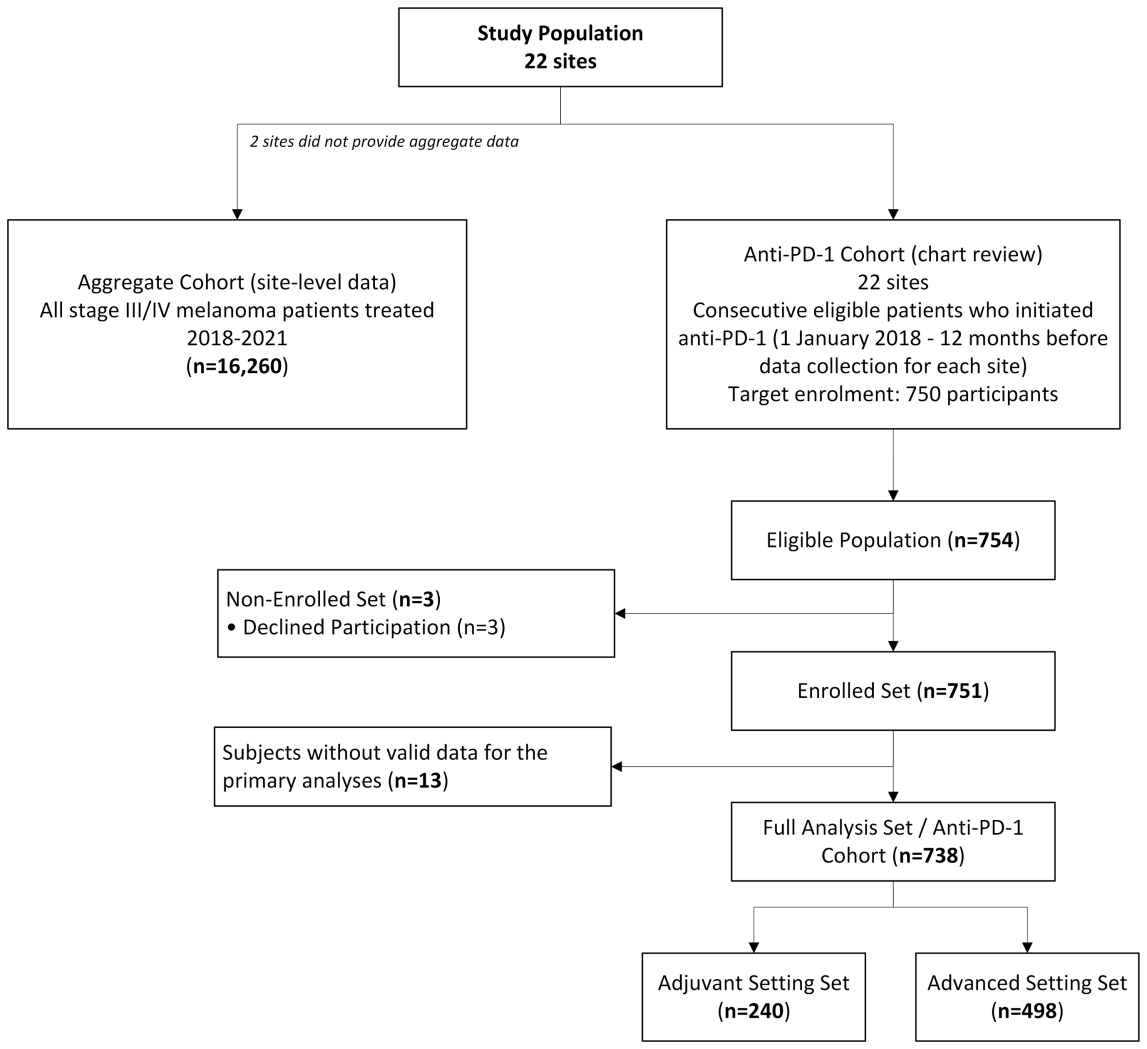
Study cohorts and patient flow. The study included an aggregate site-level cohort used to estimate treatment patterns among all stage III/IV patients managed between 2018 and 2021 and a patient-level anti-PD-1 cohort consisting of consecutively enrolled eligible patients undergoing detailed chart review analyses.

A total of 16,260 patients were included in the aggregate cohort (7935 stage III and 8325 stage IV) (**Figure 1**). As shown in **Supplementary Table 1**, patients aged <50 were more likely to present with stage III disease than stage IV (55.2% *vs.* 44.8%), whereas 54.4% of patients aged >65 had stage IV disease. In females, a higher proportion was stage III as compared to stage IV (53.6% *vs.* 46.4%), while in males, a higher percentage were stage IV as compared to stage III (54.6% *vs.* 45.4%). By country, patients in France and South Korea were predominantly stage IV, with 66.9% and 91.9% reported to have stage IV, respectively. In contrast, patients in the USA were predominantly stage III (59.8%) (**Supplementary Table 1**).

Across all countries, most stage III treatment episodes occurred in the adjuvant setting (87.6%), while most stage IV treatment episodes were managed in the advanced setting (83.7%). In the adjuvant setting, the primary management strategies for stage III were observation/watchful waiting (56.8%), anti-PD1 monotherapy (26.0%), and BRAF-targeted therapy (9.5%). In the advanced setting, the most common treatments for stage IV patients were anti-PD1 monotherapy (35.4%), anti-PD1 combination therapy (21.4%), and BRAF-targeted therapy (21.1%) (**Supplementary Table 2**). Treatment patterns varied across participating countries and regions (**Supplementary Figure 1**). In the adjuvant setting, anti-PD-1 monotherapy was most commonly reported in Germany (26.9%), followed by France (18.9%) and the UK (14.6%), whereas observation/watchful waiting was most frequently reported in the USA (56.4%). In the advanced setting, anti-PD-1 monotherapy use was highest in South Korea (51.8%) and France (48.4%), while anti-PD-1 combination therapy was most frequently reported in Germany (22.0%) and France (17.4%). For BRAF targeted therapies, the highest advanced setting use was reported in France (23.7%), with the lowest use reported in the USA (4.5%).

### 3.2 Patient characteristics in the anti-PD-1 cohort

754 patients were eligible for the anti-PD-1 cohort, of whom 3 declined participation. Thirteen patients were subsequently excluded from the enrolled set because of missing primary analysis data, yielding a full analysis set of 738 patients: 240 in the adjuvant setting (median follow-up 48.6 months) and 498 in the advanced setting (median follow-up 32.3 months) (**Figure 1**). In the adjuvant setting (**Table 1**), the mean age was 61.7 years. Patients with stage IV disease were slightly older than those with stage III (65.3 *vs.* 61.2 years). Overall, 60.8% of patients were male and most patients were White/Caucasian (82.3%). In the advanced setting (**Table 1**), the mean age was 66.5 years. Males comprised 64.5% of patients overall and were more frequently represented among patients with stage IV disease than stage III disease (65.8% vs 55.9%). White/Caucasian patients accounted for 73.7%.

**Table 1:** Demographic characteristics and disease history at index date by diagnosis stage per setting in the anti-PD-1 cohort.

Patient characteristics ^a^	Adjuvant setting			Advanced setting		
	Overall	Stage III	Stage IV	Overall	Stage III	Stage IV
	(N = 240)	(N = 211)	(N = 29)	(N = 498)	(N = 68)	(N = 430)
Age at index date (years)						
Mean (SD)	61.7 (14.6)	61.2 (14.8)	65.3 (12.4)	66.5 (14.2)	67.9 (15.2)	66.3 (14.1)
Gender						
Male	146 (60.8)	126 (59.7)	20 (69.0)	321 (64.5)	38 (55.9)	283 (65.8)
Female	94 (39.2)	85 (40.3)	9 (31.0)	177 (35.5)	30 (44.1)	147 (34.2)
BMI						
BMI <25	55 (22.9)	51 (24.2)	4 (13.8)	139 (27.9)	11 (16.2)	128 (29.8)
25≤ BMI <30	82 (34.2)	71 (33.6)	11 (37.9)	141 (28.3)	22 (32.4)	119 (27.7)
BMI ≥30	59 (24.6)	56 (26.5)	3 (10.3)	109 (21.9)	16 (23.5)	93 (21.6)
Unknown	44 (18.3)	33 (15.6)	11 (37.9)	109 (21.9)	19 (27.9)	90 (20.9)
Race						
White/Caucasian	163 (82.3)	143 (83.6)	20 (74.1)	306 (73.7)	42 (73.7)	264 (73.7)
Asian	13 (6.6)	13 (7.6)	0 (0.0)	37 (8.9)	0 (0.0)	37 (10.3)
Others	1 (0.5)	1 (0.6)	0 (0.0)	8 (2.0)	3 (5.2)	5 (1.5)
Unknown	21 (10.6)	14 (8.2)	7 (25.9)	64 (15.4)	12 (21.1)	52 (14.5)
Missing^	42	40	2	83	11	72
Performance status measured						
Unknown	22 (9.2)	13 (6.2)	9 (31.0)	33 (6.6)	6 (8.8)	27 (6.3)
No	26 (10.8)	21 (10.0)	5 (17.2)	57 (11.4)	3 (4.4)	54 (12.6)
Yes	192 (80)	177 (83.9)	15 (51.7)	408 (81.9)	59 (86.8)	349 (81.2)
ECOG						
0	145 (75.5)	134 (75.7)	11 (73.3)	225 (55.6)	36 (61.0)	189 (54.6)
1	46 (24)	42 (23.7)	4 (26.7)	147 (36.3)	16 (27.1)	131 (37.9)
2	1 (0.5)	1 (0.6)	0 (0.0)	22 (5.4)	5 (8.5)	17 (4.9)
≥ 3	0 (0)	0 (0.0)	0 (0.0)	11 (2.7)	2 (3.4)	9 (2.6)
Missing ECOG^b^	0	0	0	3	0	3
CCI score						
0	23 (9.6)	23 (10.9)	0 (0.0)	0 (0.0)	0 (0.0)	0 (0.0)
1	4 (1.7)	4 (1.9)	0 (0.0)	0 (0.0)	0 (0.0)	0 (0.0)
2	1 (0.4)	1 (0.5)	0 (0.0)	0 (0.0)	0 (0.0)	0 (0.0)
≥ 3	212 (88.3)	183 (86.7)	29 (100.0)	498 (100.0)	68 (100.0)	430 (100.0)
Lymphedema						
Unknown	36 (15.0)	28 (13.2)	8 (27.6)	55 (11.0)	6 (8.8)	49 (11.3)
No	186 (77.5)	166 (78.7)	20 (69.0)	406 (81.5)	54 (79.4)	352 (81.9)
Yes	18 (7.5)	17 (8.1)	1 (3.4)	37 (7.4)	8 (11.8)	29 (6.7)
Comorbid condition(s)						
Unknown	4 (1.7)	4 (1.9)	0 (0.0)	8 (1.6)	1 (1.5)	7 (1.6)
No	122 (50.8)	106 (50.2)	16 (55.2)	224 (45.0)	27 (39.7)	197 (45.8)
Yes	114 (47.5)	101 (47.9)	13 (44.8)	266 (53.4)	40 (58.8)	226 (52.6)

BMI, body mass index; CCI, Charlson Comorbidity Index; ECOG, Eastern Cooperative Oncology Group; SD, Standard Deviation.

^a^ All data represented by N and %, except otherwise indicated.

^b^ Percentages for Missing values are not applicable and therefore not reported. “Missing” indicates that the data field was not completed.

### 3.3 Index LOT characteristics in the anti-PD-1 cohort

In the adjuvant setting (**Table 2**), 97.5% of patients received anti-PD-1 as monotherapy, including all stage III and most stage IV patients, while 20.7% with stage IV received it as combination therapy. The mean duration of therapy was 290.7 days (SD 136.1), longer in stage III than stage IV (298.6 *vs.* 233.0 days). The most common reason for permanent discontinuation was completion of planned therapy (46.0%), followed by disease progression (20.5%). The median time to permanent discontinuation of the index anti-PD-1 therapy was 337 days (Q1: 212.0, Q3: 365.0) (**Supplementary Figure 2A**). Persistence was 88.3% at 3 months, 77.5% at 6 months, 68.7% at 9 months, and 27.2% at 12 months.

**Table 2:** Index LOT characteristics by diagnosis stage per setting in the full analysis set (FAS) of anti-PD-1 cohort.

Index lOT characteristics ^a^	Adjuvant setting			Advanced setting		
	Overall	Stage III	Stage IV	Overall	Stage III	Stage IV
	(N = 240)	(N = 211)	(N = 29)	(N = 498)	(N = 68)	(N = 430)
Treatment regime						
Anti-PD-1 Monotherapy	234 (97.5)	211 (100.0)	23 (79.3)	314 (63.1)	47 (69.1)	267 (62.1)
Anti-PD-1 + Anti-CTLA-4	6 (2.5)	0 (0.0)	6 (20.7)	179 (35.9)	20 (29.4)	159 (37.0)
Anti-PD-1 + Others	0 (0.0)	0 (0.0)	0 (0.0)	5 (1.0)	1 (1.5)	4 (0.9)
Anti-PD-1 duration (days)						
Mean (SD)	290.7 (136.1)	298.6 (133.4)	233.0 (144.4)	223.2 (277.5)	203.2 (226.9)	226.3 (284.7)
Temporary interruption						
Unknown	14 (5.8)	14 (6.6)	0 (0.0)	17 (3.4)	2 (2.9)	15 (3.5)
No	184 (76.7)	160 (75.8)	24 (82.8)	386 (77.5)	54 (79.4)	332 (77.2)
Yes	42 (17.5)	37 (17.5)	5 (17.2)	95 (19.1)	12 (17.6)	83 (19.3)
Concomitant medications						
Unknown	28 (11.7)	21 (10.0)	7 (24.1)	36 (7.2)	7 (10.3)	29 (6.7)
No	154 (64.2)	141 (66.8)	13 (44.8)	277 (55.6)	37 (54.4)	240 (55.8)
Yes	58 (24.2)	49 (23.2)	9 (31.0)	185 (37.1)	24 (35.3)	161 (37.4)
Permanent interruption						
No	2 (0.8)	1 (0.5)	1 (3.4)	18 (3.6)	1 (1.5)	17 (4.0)
Yes	238 (99.2)	210 (99.6)	28 (96.5)	480 (96.4)	67 (98.6)	413 (96.1)
Reasons for permanent discontinuation						
Completed planned therapy	110 (46.0)	99 (46.9)	11 (39.3)	73 (14.9)	12 (17.6)	61 (14.5)
Disease progression	49 (20.5)	46 (21.8)	3 (10.7)	166 (33.9)	21 (30.9)	145 (34.4)
Complete response	17 (7.1)	16 (7.6)	1 (3.6)	42 (8.6)	8 (11.8)	34 (8.1)
Doctor decision	36 (15.1)	28 (13.3)	8 (28.6)	115 (23.5)	13 (19.1)	102 (24.2)
Patient decision	10 (4.2)	7 (3.3)	3 (10.7)	26 (5.3)	2 (2.9)	24 (5.7)
Palliative care	1 (0.4)	1 (0.5)	0 (0.0)	9 (1.8)	1 (1.5)	8 (1.9)
Death	0 (0.0)	0 (0.0)	0 (0.0)	9 (1.8)	3 (4.4)	6 (1.4)
Others	18 (7.5)	15 (7.1)	3 (10.7)	75 (15.3)	8 (11.8)	67 (15.9)
Unknown	3 (1.3)	1 (0.5)	2 (7.1)	6 (1.2)	2 (2.9)	4 (1.0)

SD, Standard deviation.

^a^ All data represented by N and %, except otherwise indicated.

In the advanced setting (**Table 2**), 63.1% of patients received anti-PD-1 as monotherapy and 36.9% received it as combination therapy, predominantly anti-PD-1 plus anti-CTLA-4 (35.9%). By stage, monotherapy was given to 69.1% of stage III patients and 62.1% of stage IV patients, while combination therapy was used in 30.9% of stage III and 37.9% of stage IV patients, respectively. The mean duration of therapy was 223.2 days (SD 277.5), with similar durations across stages. Disease progression was the leading cause of permanent discontinuation (33.9%), followed by doctor decision (23.1%) and completion of planned therapy (14.9%). The median time to permanent discontinuation of the index anti-PD-1 therapy was 94.0 days (Q1: 50.0, Q3: 331.0) (**Supplementary Figure 2B**). Persistence was 50.8% at 3 months, 37.3% at 6 months, 27.9% at 9 months, and 21.3% at 12 months.

### 3.4 Anti-melanoma therapies before and after disease progression in the anti-PD-1 cohort

Adjuvant setting (**Table 3**): Before the index date, surgery was reported in 99.2% of patients, while radiotherapy was given to 7.1%, and systemic therapy to 0.9% of patients. After the index date and before progression, no surgery or radiotherapy was performed, while systemic therapy was received by 47.1% of patients. Following progression, surgery was performed in 37.2% of patients, radiotherapy was administered to 42.5% of patients, and systemic therapy given to 77.9% of patients (**Table 3**).

**Table 3:** Anti-melanoma therapies before and after disease progression by stage per setting in the anti-PD-1 cohort.

Anti-melanoma therapies, N (%)	Adjuvant Setting			Advanced Setting		
	Overall	Stage III	Stage IV	Overall	Stage III	Stage IV
	(N = 240)	(N = 211)	(N = 29)	(N = 498)	(N = 68)	(N = 430)
Before index date						
Surgery						
No	2 (0.8)	2 (0.9)	0 (0.0)	260 (52.2)	41 (60.3)	219 (50.9)
Yes	238 (99.2)	209 (99.1)	29 (100.0)	238 (47.8)	27 (39.7)	211 (49.1)
Radiotherapy						
Unknown	0 (0.0)	0 (0.0)	0 (0.0)	1 (0.2)	0 (0.0)	1 (0.2)
No	223 (92.9)	195 (92.4)	28 (96.6)	407 (81.7)	61 (89.7)	346 (80.5)
Yes	17 (7.1)	16 (7.6)	1 (3.4)	90 (18.1)	7 (10.3)	83 (19.3)
Systemic therapy						
Unknown	3 (1.3)	3 (1.4)	0 (0.0)	4 (0.8)	0 (0.0)	4 (0.9)
No	235 (97.9)	208 (98.6)	27 (93.1)	462 (92.8)	65 (95.6)	397 (92.3)
Yes	2 (0.8)	0 (0.0)	2 (6.9)	32 (6.4)	3 (4.4)	29 (6.7)
After index date and before disease progression ^a^						
Surgery						
Unknown	1 (0.4)	0 (0.0)	1 (3.4)	2 (0.4)	0 (0.0)	2 (0.5)
No	239 (99.6)	211 (100.0)	28 (96.6)	496 (99.6)	68 (100.0)	428 (99.5)
Yes	0 (0.0)	0 (0.0)	0 (0.0)	0 (0.0)	0 (0.0)	0 (0.0)
Radiotherapy						
Unknown	1 (0.4)	1 (0.5)	0 (0.0)	5 (1.0)	0 (0.0)	5 (1.2)
No	239 (99.6)	210 (99.5)	29 (100.0)	490 (98.4)	68 (100.0)	422 (98.1)
Yes	0 (0.0)	0 (0.0)	0 (0.0)	3 (0.6)	0 (0.0)	3 (0.7)
Systemic therapy (other than the index LOT)						
No	127 (52.9)	111 (52.6)	16 (55.2)	167 (33.5)	26 (38.2)	141 (32.8)
Yes	113 (47.1)	100 (47.4)	13 (44.8)	331 (66.5)	42 (61.8)	289 (67.2)
After disease progression ^b^						
Surgery						
Unknown	1 (0.9)	0 (0.0)	1 (7.7)	2 (0.6)	0 (0.0)	2 (0.7)
No	70 (61.9)	60 (60.0)	10 (76.9)	266 (79.2)	32 (74.4)	234 (79.9)
Yes	42 (37.2)	40 (40.0)	2 (15.4)	68 (20.2)	11 (25.6)	57 (19.4)
Radiotherapy						
Unknown	0 (0.0)	0 (0.0)	0 (0.0)	5 (1.5)	0 (0.0)	5 (1.7)
No	65 (57.5)	55 (55.0)	10 (76.9)	187 (55.7)	26 (60.5)	161 (54.9)
Yes	48 (42.5)	45 (45.0)	3 (23.1)	144 (42.9)	17 (39.5)	127 (43.3)
Systemic therapy (other than the index LOT)						
No	25 (22.1)	19 (19.0)	6 (46.1)	129 (38.4)	16 (37.2)	113 (38.6)
Yes	88 (77.9)	81 (81.0)	7 (53.9)	207 (61.6)	27 (62.8)	180 (61.4)

^a^ Treatments reported after the index date refer to any other treatment distinct from those comprising the index LOT.

^b^ Only applicable to patients with disease progression.

Advanced setting (**Table 3**): Prior to the index date, surgery was performed in 47.8% of patients, radiotherapy was administered to 18.1% of patients, and systemic therapy was given to 6.4% of patients. After the index date and before progression, no surgery was performed and only 0.7% of patients received radiotherapy, while systemic therapy was received by 66.5%. Following progression, surgery was performed in 20.2% of patients, radiotherapy was administered to 42.9%, and systemic therapy was given to 61.6% of patients (**Table 3**).

### 3.5 Index LOT characteristics in patients with or without disease progression in the anti-PD-1 cohort

In the adjuvant setting (**Table 4**), anti-PD-1 monotherapy was administered to 53.4% of patients with disease progression and 46.6% of those without progression during the study time frame, while anti-PD-1 plus anti-CTLA-4 was given to 33.3% of patients without progression and 66.7% with progression during the study time frame (p = 0.424). The mean duration of anti-PD-1 therapy in the index LOT was 305.9 days (SD 98.7) for patients without progression and 273.6 days (SD 167.4) for those with progression (p = 0.005) (median follow-up 48.6 months).

**Table 4:** Index LOT characteristics in anti-PD-1 cohort patients with or without disease progression.

Index LOT characteristics ^a^	Adjuvant setting (N = 240)			Advanced setting (N = 498)		
	Patients without progression (N = 127)	Patients with progression (N = 113)	P value	Patients without progression (N = 162)	Patients with progression (N = 336)	P value
Treatment regime						
Anti-PD-1 Monotherapy	125 (53.4)	109 (46.6)	0.424	92 (29.3)	222 (70.7)	0.059
Anti-PD-1 + Anti-CTLA-4	2 (33.3)	4 (66.7)		67 (37.4)	112 (62.6)	
Anti-PD-1 + Others	0 (0.0)	0 (0.0)		3 (60.0)	2 (40.0)	
Anti-PD-1 duration (days)						
Mean (SD)	305.9 (98.7)	273.6 (167.4)	0.005	253.6 (270.5)	208.5 (279.9)	0.194
Temporary interruption						
Unknown	6 (42.9)	8 (57.1)	0.510	7 (41.2)	10 (58.8)	0.145
No	96 (52.2)	88 (47.8)		117 (30.3)	269 (69.7)	
Yes	25 (59.5)	17 (40.5)		38 (40.0)	57 (60.0)	
Concomitant medications						
Unknown	13 (46.4)	15 (53.6)	0.525	6 (16.7)	30 (83.3)	0.103
No	80 (51.9)	74 (48.1)		95 (34.3)	182 (65.7)	
Yes	34 (58.6)	24 (41.4)		61 (33.0)	124 (67.0)	

SD, Standard deviation.

^a^ All data represented by N and %, except otherwise indicated.

In the advanced setting (**Table 4**), anti-PD-1 monotherapy was administered to 29.3% of patients with progression and 70.7% of those without progression during the study time frame, while anti-PD-1 plus anti-CTLA-4 was given to 37.4% patients with progression and 62.6% of those without progression during the study time frame (p = 0.06). Mean anti-PD-1 duration was 253.6 days (SD 270.5) among patients without progression and 208.5 days (SD 279.9) among those with progression (p = 0.194) (median follow-up 32.3 months).

### 3.6 Treatment patterns across successive LOTs in the anti-PD-1 cohort

**Figure 2:**
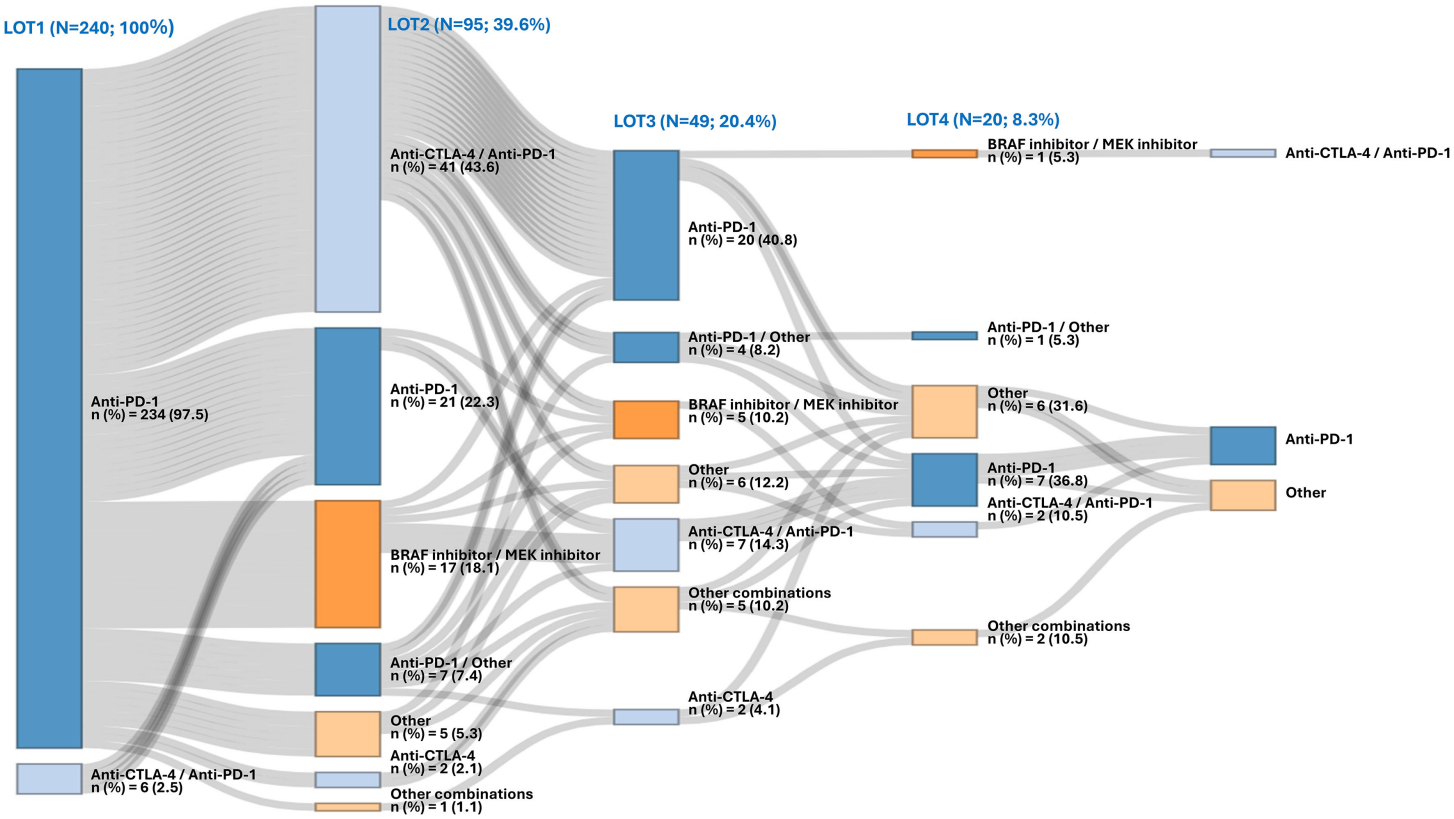
Sequence of lines of therapy (LOTs) in the adjuvant setting. Only patients who proceeded to the corresponding LOT are represented.

Treatment pattern analyses for each LOT included only patients who proceeded to the corresponding subsequent LOT. As expected, the number of evaluable patients contributing to each LOT analysis decreased substantially across successive LOTs because of attrition (**Supplementary Tables 3**, **4**). For the adjuvant setting (**Supplementary Table 3**; **Figure 2**), characteristics of the first (index) LOT are described in detail in section 3.4. Anti-PD-1-based regimens remained commonly used across successive LOTs in the adjuvant setting. Among patients who proceeded to a second LOT in the adjuvant anti-PD-1 cohort (N = 95), anti-PD-1 monotherapy accounted for 22.3% and combination therapy 70.2%, including anti-PD-1 plus anti-CTLA-4 (43.6%) and BRAF/MEK inhibitor therapy (18.1%). Among those patients who received a third LOT (N = 49), 40.8% received anti-PD-1 monotherapy and 42.9% received combination regimens, including anti-PD-1 plus anti-CTLA-4 (14.3%) and other combinations (10.2%). Of those patients who received a fourth LOT (N = 20), anti-PD-1 monotherapy was given to 36.8% while 30.0% received combination therapy. The mean duration of therapy was 290.7 days (SD 136.1), 179.2 days (SD 253.2), 168.6 days (SD 166.8), and 153.2 days (SD 256.2) in the first, second, third, and fourth LOT, respectively.

**Figure 3:**
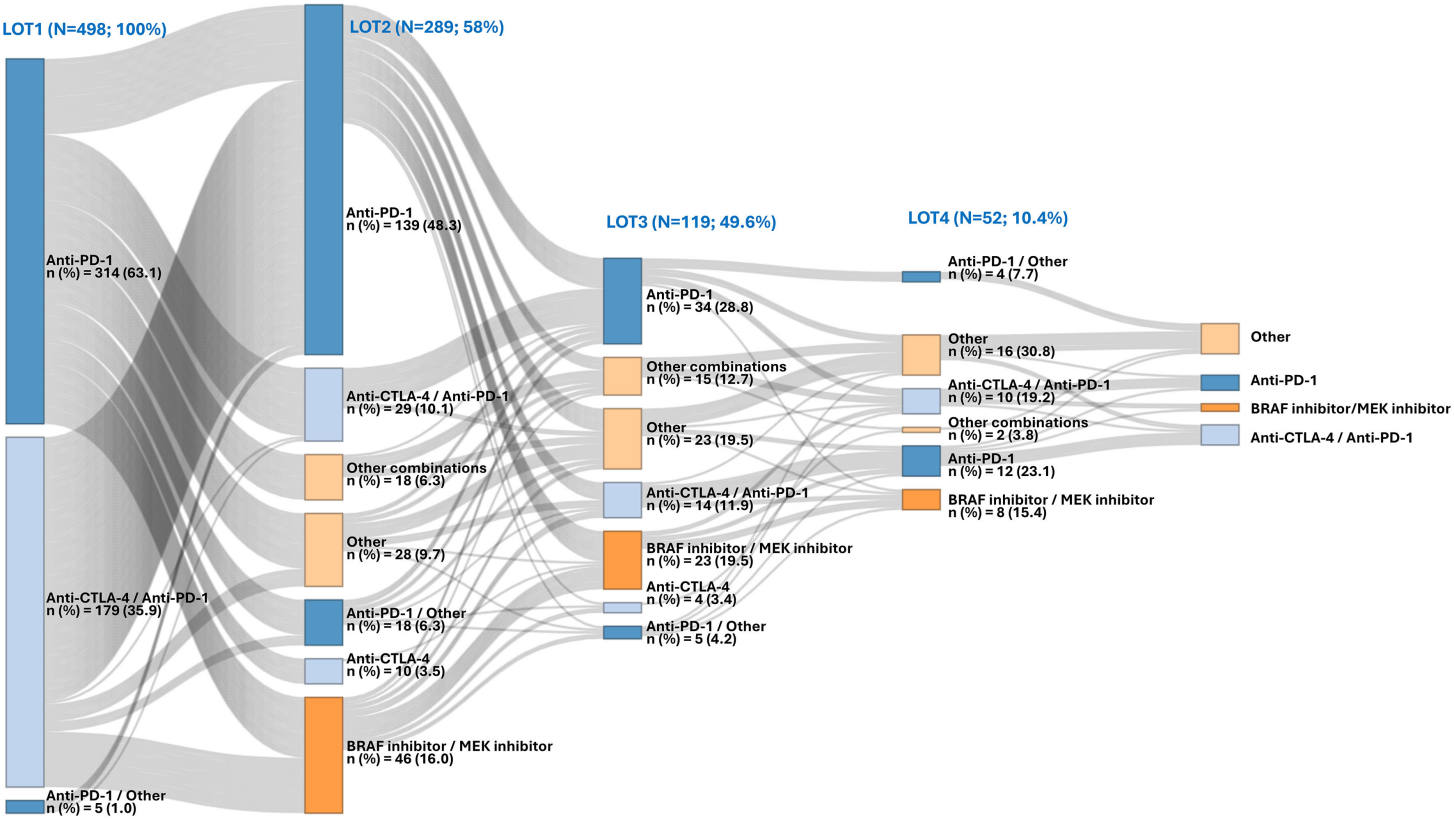
Sequence of lines of therapy in the advanced setting. Only patients who proceeded to the corresponding LOT are represented.

For the advanced setting (**Supplementary Table 4**; **Figure 3**), characteristics of the index LOT are as described in section 3.4. Anti-PD-1 monotherapy and anti-PD-1-based combination regimens continued to be used as across later LOTs in the advanced setting. Among patients who received a second LOT (N = 289), 48.3% were treated with anti-PD-1 monotherapy, and 9.7% received other monotherapies. Combination therapy was used to treat 38.4%, including 10.1% who received anti-PD-1 plus anti-CTLA-4 and 16.0% who were treated with BRAF plus MEK inhibitors. Of those patients who received a third LOT (N = 119), 28.8% were treated with anti-PD-1 monotherapy, and 19.5% received other monotherapies. Combination therapy was used to treat 47.9% of patients, including 11.9% who received anti-PD-1 plus anti-CTLA-4 and 19.5% who received BRAF plus MEK inhibitors. Among those patients who received a fourth LOT (N = 52), 23.1% were treated with anti-PD-1 monotherapy, and 30.8% received other monotherapies. Combination therapy was used to treat 46.2% of patients, including 19.2% treated with anti-PD-1 plus anti-CTLA-4 and 15.4% who received BRAF plus MEK inhibitors. The mean duration of therapy was 223.2 days (SD 277.5), 272.7 days (SD 344.3), 222.6 days (SD 310.9), and 163.3 days (SD 245.9) in the first, second, third, and fourth LOT, respectively.

## 4 Discussion

This multinational real-world study characterized treatment utilization and sequencing patterns for patients with stage III-IV melanoma in both the adjuvant and advanced settings, with a focus on patients receiving anti-PD-1-based therapies. These data underscore the central role of anti-PD-1 therapies across disease stages, while revealing setting-specific differences in treatment duration, sequencing, and reasons for discontinuation – patterns that both align with and extend the existing literature. The frequent reuse of anti-PD-1 therapy in both settings confirms its established position in melanoma management and reinforces the need for novel therapeutic options, evidence-based sequencing strategies, and multidisciplinary care models to improve long-term outcomes.

In the aggregate cohort, variation in systemic therapy use was revealed across the disease stages. For resected stage III melanoma, observation was the most common management approach, with just over one-quarter of patients receiving anti-PD-1 therapy and fewer still receiving BRAF-targeted therapy. As stage III disease is heterogeneous, the observed treatment rates may in part reflect the underlying distribution of stage III subcategories, particularly the proportion of patients with lower-risk stage IIIA disease (18). These findings illustrate the limited adoption of adjuvant systemic therapy, consistent with NCCN guidelines that permit observation in selected patients (19). In contrast, a US-based study of stage III cutaneous melanoma reported broader adoption of adjuvant anti-PD-1 therapy, with utilization influenced by disease substage, insurance coverage, and performance status (20).

In the advanced setting, systemic therapy predominated for stage IV disease, with anti-PD-1 monotherapy and combination therapy representing the most common approaches. These patterns align with prior US reports in which first-line regimens often comprised anti-PD-1 monotherapy, ipilimumab-based regimens, and BRAF/MEK inhibitors (17) reflecting the influence of pivotal clinical trials that established the efficacy of immunotherapy and targeted therapy, and subsequently informed guideline recommendations and clinical practice standards (8).

Notably, the distribution of stage III and stage IV disease varied across participating countries, with some countries (France and South Korea) contributing proportionally larger stage IV populations. Differences in treatment patterns were also observed across participating countries and regions. For example, greater use of anti-PD-1 monotherapy in the advanced setting was observed in France and South Korea, whereas higher proportions of observation/watchful waiting in the adjuvant setting were observed in the USA. These differences likely reflect multiple factors, including variation in healthcare systems, reimbursement and access pathways, timing of the incorporation of newer therapies into clinical practice, local treatment guidelines, and underlying patient or disease characteristics (21, 22). Additionally, regional differences in melanoma subtype epidemiology, such as the higher prevalence of acral melanoma in Asian populations, which is more often diagnosed at more advanced stages, may also contribute to such variability (23).

In the main anti-PD-1 cohort with chart-review which provided more granular clinical data from patients who received anti-PD-1 therapy, treatment patterns largely aligned with clinical guidelines (19), with near-universal use of anti-PD-1 in monotherapy among stage III patients. Reasons for discontinuation differed between the adjuvant and advanced settings, with treatment completion dominating in adjuvant disease and progression dominating in advanced disease. In the adjuvant setting, median treatment duration was approximately 11 months (median follow-up ~49 months), and discontinuation was most often due to planned completion, suggesting more favourable outcomes. While randomized clinical trials typically prescribe a 12-month course of adjuvant immunotherapy (24), published real-world data report frequent premature discontinuation, often due to toxicity (25). In this regard, there is uncertainty regarding the optimal treatment duration in the adjuvant setting. In our study, patients without progression had significantly longer mean treatment durations than those with progression, although shorter treatment duration may also simply reflect earlier progression – the present study was not designed to compare outcomes between observation and adjuvant anti-PD-1 strategies, and treatment duration should not be interpreted as a surrogate for comparative clinical benefit. Some studies suggest that shorter treatment durations may not negatively affect outcomes, while minimizing toxicity and cost (24, 26). Ongoing and future clinical trials are critical to address these questions.

Treatment sequencing patterns in the adjuvant setting emphasize the clinical challenges faced after recurrence. While second-line treatment often involved anti-PD1 monotherapy, later lines showed greater heterogeneity but progressively shorter treatment durations, highlighting reduced benefit/tolerability and reinforcing the need for therapeutic innovations for those patients who progress following adjuvant anti-PD-1 therapy. Current treatment options for this population are limited, with little evidence to guide subsequent treatments after progression, particularly for those patients who progress early or without having received prior targeted therapy.

In the advanced setting, anti-PD-1 monotherapy predominated as the index LOT, while a substantial minority received combination anti-PD-1 plus anti-CTLA-4. The median treatment duration was short (~3 months), and discontinuation was largely driven by disease progression, reflecting the aggressive nature of unresectable disease and the greater need for subsequent therapy. Although no consensus exists regarding optimal treatment duration for advanced melanoma in clinical practice (27, 28) and data on treatment patterns among patients who received anti-PD-1 are limited, our observations are broadly consistent with prior reports. Mäkelä et al. (29) implemented a six-month anti-PD-1 treatment program in which only 17 of 38 patients completed the full duration. Kartolo et al. (30) similarly observed a median treatment duration of 3 months among patients who discontinued anti-PD-1 therapy due to progression. These patterns collectively reinforce the challenges of maintaining long-term therapy in this population.

In the advanced setting, surgical management was infrequent before the index date, consistent with unresectable disease, and systemic therapy was the mainstay both before and after progression. Treatment durations did not differ substantially by progression status, possibly reflecting earlier discontinuation due to intolerance in both groups. Treatment sequencing patterns further illustrate the limitations in treatment options: anti-PD-1 monotherapy predominated across multiple lines, with BRAF/MEK inhibitors and checkpoint inhibitor combinations becoming more frequent after the second LOT. Sample sizes declined substantially beyond LOT2 because of expected attrition across successive therapy lines in this real-world cohort, consistent with prior reports indicating that more than 40% of advanced patients do not receive a second LOT after progression (31, 32). Taken together, these findings emphasize two key aspects relating to advanced melanoma. First, effective options after progression on anti-PD-1 therapy are limited (particularly for BRAF-wild-type patients), which likely contributes to high attrition after the index LOT. Second, the frequent reuse of immunotherapy beyond progression suggests ongoing uncertainty about the benefit of rechallenge or continuation strategies. Addressing these gaps will require prospective studies to clarify the value of retreatment, optimize sequencing, and identify subgroups most likely to benefit from specific post-progression approaches. The high rates of progression-related discontinuation, short treatment durations, substantial attrition across LOTs, and continued reuse of anti-PD-1-based regimens suggest an ongoing unmet need for effective therapies after progression on anti-PD-1 treatment.

An additional clinically relevant subgroup includes patients with recurrence or progression after anti-PD-1 therapy who are subsequently managed with local treatment approaches alone, such as surgery or radiotherapy, without further systemic therapy. Although surgery and radiotherapy were collected in the present study, the dataset was not specifically structured to robustly identify patients managed exclusively with local therapy following recurrence/progression. Further investigation of this subgroup in future real-world studies would be of interest.

This study has several notable strengths. The analysis leverages a large, multinational real-world dataset that included both adjuvant and advanced melanoma, enhancing the generalizability of findings across diverse clinical settings. The use of a contemporaneous background cohort from the same sites provides valuable context for interpreting treatment uptake and sequencing. Stage-specific analyses enabled clear contrasts between adjuvant and advanced settings, while detailed persistence and sequencing evaluations offer novel insights into treatment trajectories rarely captured outside clinical trials. Nonetheless, the retrospective design introduces risks of missing data and selection bias, and small sample sizes in later LOT constrain the robustness of inferences for those subgroups. Further, this dataset includes patients treated between 2018 and 2021 and thus does not reflect the use of nivolumab/relatlimab combination that became available in 2022. Similarly, the predominance of White/Caucasian patients in several participating regions may also limit generalizability to more diverse populations. This distribution may, at least in part, reflect the epidemiology of the disease and the regions participating in the study. Future studies should aim to include more diverse patient populations to improve the generalizability of real-world evidence across different racial and ethnic groups.

This multinational real-world analysis provides important insights into the evolving landscape of melanoma management, highlighting the central and sustained role of anti-PD-1 therapies in patients with stage III-IV disease. The findings reveal heterogeneity in systemic therapy utilization, treatment duration, and sequencing, reflecting both adherence to clinical guidelines and variability in real-world practice. Treatment patterns after progression suggest limited therapeutic options, and in the absence of effective alternative options, retreatment with anti-PD-1 agents is frequently employed in subsequent LOTs. Further research is needed to develop therapeutic strategies for this population with limited post-anti-PD-1 treatment options.

## Data Availability

The original contributions presented in the study are included in the article/**Supplementary Material**, further inquiries can be directed to the corresponding author.
