# Supplementary material for: Real-world treatment patterns in stage III and IV melanoma patients: insights from a global multi-center chart review study
Source: Front Immunol. 2026 Jul 23;17:1813387. doi: 10.3389/fimmu.2026.1813387 (PMC13442371; doi:10.3389/fimmu.2026.1813387)
Supplement: Supplementary file 1 [file Table1.docx]

Supplementary Table 1. Aggregated cohort (cumulative site-level data): Patient characteristics by stage

| Patients Characteristics | **Stage III**  (N = 7935) | **Stage IV**  (N= 8325) |
| --- | --- | --- |
| Age |  |  |
| < 50 years | 1722 (55.2) | 1396 (44.8) |
| 50-65 years | 2837 (49.1) | 2943 (50.9) |
| > 65 years | 3375 (45.9) | 3979 (54.1) |
| Unknown^*^ | 1 | 7 |
| Sex |  |  |
| Female | 3662 (53.6) | 3176 (46.4) |
| Male | 4273 (45.4) | 5147 (54.6) |
| Unknown^*^ | 0 | 2 |
| Country |  |  |
| France | 888 (33.1) | 1794 (66.9) |
| Germany | 1159 (44.9) | 1420 (55.1) |
| UK | 668 (42.5) | 905 (57.5) |
| USA | 4207 (59.8) | 2824 (40.2) |
| Australia | 982 (48.8) | 1029 (51.2) |
| South Korea | 31 (8.1) | 353 (91.9) |

^*^Percentages for Unknown values categories are not applicable and therefore not reported.

Supplementary Table 2. Aggregated cohort (cumulative site-level data): Treatments by stage per setting

| Treatments^*^ | **Stage III**  (N = 7935) | **Stage IV**  (N= 8325) |
| --- | --- | --- |
| *Adjuvant setting* |  |  |
| Observation/watchful waiting | 4498 (56.8) | 1139 (13.7) |
| Anti-PD-1 monotherapy | 2056 (26.0) | 328 (3.9) |
| BRAF targeted therapy | 750 (9.5) | 84 (1.0) |
| Other | 159 (2.0) | 25 (0.3) |
| Unknown^**^ | 22 | 0 |
| *Advanced setting* |  |  |
| Anti-PD-1 monotherapy | 432 (5.5) | 2942 (35.4) |
| Anti-PD-1 based combination therapy | 237 (3.0) | 1775 (21.4) |
| BRAF targeted therapy | 163 (2.1) | 1752 (21.1) |
| Other | 224 (2.8) | 1622 (19.5) |
| Unknown^**^ | 12 | 23 |

^*^Percentages for each treatment were calculated based on the total number of patients reported in that stage with a known treatment (regardless of the type of setting). Percentages total could be over 100% because some patients may have received more than one treatment categories.

^**^Percentages for Unknown values categories are not applicable and therefore not reported.

**Supplementary Table 3.** Treatment Patterns by LOT in the Adjuvant Anti-PD-1 Cohort, with LOT-Specific Denominators

| **Treatment Patterns** | **First LOT** | **Second LOT** | **Third LOT** | **Fourth LOT** |
| --- | --- | --- | --- | --- |
|  | **(N=240)** | **(N=95)** | **(N=49)** | **(N=20)** |
| **LOT Composition** |  |  |  |  |
| ***Monotherapy*** | ***234 (97.5)*** | ***28 (29.5)*** | ***28 (57.1)*** | ***13 (65.0)*** |
| Anti-PD1 | 234 (97.5) | 21 (22.3) | 20 (40.8) | 7 (36.8) |
| Anti-CTLA -4 | 0 (0.0) | 2 (2.1) | 2 (4.1) | 0 (0.0) |
| Other | 0 (0.0) | 5 (5.3) | 6 (12.2) | 6 (31.6) |
| ***Combination Therapy*** | ***6 (2.5)*** | ***66 (69.5)*** | ***21 (42.9)*** | ***6 (30.0)*** |
| Anti-PD-1 + Anti-CTLA-4 | 6 (2.5) | 41 (43.6) | 7 (14.3) | 2 (10.5) |
| BRAF inhibitor + MEK inhibitor | 0 (0.0) | 17 (18.1) | 5 (10.2) | 1 (5.3) |
| Anti-PD1 + Other(s) | 0 (0.0) | 7 (7.4) | 4 (8.2) | 1 (5.3) |
| Other Combinations | 0 (0.0) | 1 (1.1) | 5 (10.2) | 2 (10.5) |
| **Duration of LOT (days)** |  |  |  |  |
| N, missing | 240, 0 | 94, 1 | 48, 1 | 20, 0 |
| Mean (SD) | 290.7 (136.1) | 179.2 (253.2) | 168.6 (166.8) | 153.2 (256.2) |
| **Drug part of Clinical Trial** |  |  |  |  |
| No | 6 (2.5) | 141 (148.4) | 60 (122.4) | 21 (105.0) |
| Yes | 0 (0.0) | 24 (25.3) | 12 (24.5) | 5 (25.0) |
| **Concomitant medications per LOT** |  |  |  |  |
| Unknown | 28 (11.7) | 12 (17.4) | 4 (12.9) | 1 (10.0) |
| No | 154 (64.2) | 36 (52.2) | 18 (58.1) | 6 (60.0) |
| Yes | 58 (24.2) | 21 (30.4) | 9 (29.0) | 3 (30.0) |

Supplementary Table 4. Treatment Patterns by LOT in the Advanced Anti-PD-1 Cohort, with LOT-Specific Denominators

| **Treatment Patterns** | **First LOT** | **Second LOT** | **Third LOT** | **Fourth LOT** |
| --- | --- | --- | --- | --- |
|  | **(N=498)** | **(N=289)** | **(N=119)** | **(N=52)** |
| **LOT Composition** |  |  |  |  |
| ***Monotherapy*** | *314 (63.1)* | *177 (61.2)* | *61 (51.3)* | *28 (53.8)* |
| Anti-PD-1 | 314 (63.1) | 139 (48.3) | 34 (28.8) | 12 (23.1) |
| Anti-CTLA-4 | 0 (0.0) | 10 (3.5) | 4 (3.4) | 0 (0.0) |
| Other | 0 (0.0) | 28 (9.7) | 23 (19.5) | 16 (30.8) |
| ***Combination*** | *184 (36.9)* | *111 (38.4)* | *57 (47.9)* | *24 (46.2)* |
| Anti-PD-1+CTLA-4 | 179 (35.9) | 29 (10.1) | 14 (11.9) | 10 (19.2) |
| BRAF inhibitor + MEK inhibitor | 0 (0.0) | 46 (16.0) | 23 (19.5) | 8 (15.4) |
| Anti-PD-1 + Other(s) | 5 (1.0) | 18 (6.3) | 5 (4.2) | 4 (7.7) |
| Other Combinations | 0 (0.0) | 18 (6.3) | 15 (12.7) | 2 (3.8) |
| **Duration of LOT (days)** |  |  |  |  |
| N, missing | 498, 0 | 287, 2 | 119, 0 | 52, 0 |
| Mean (SD) | 223.2 (277.5) | 272.7 (344.3) | 222.6 (310.9) | 163.3 (245.9) |
| **Drug part of Clinical Trial** |  |  |  |  |
| Unknown | 4 (0.8) | 6 (2.1) | 6 (5.0) | 1 (1.9) |
| No | 169 (33.9) | 364 (126.0) | 155 (130.3) | 71 (136.5) |
| Yes | 11 (2.2) | 43 (14.9) | 22 (18.5) | 6 (11.5) |
| **Concomitant medications per LOT** |  |  |  |  |
| Unknown | 36 (7.2) | 14 (7.5) | 3 (5.7) | 1 (3.8) |
| No | 277 (55.6) | 100 (53.5) | 25 (47.2) | 15 (57.7) |
| Yes | 185 (37.1) | 73 (39.0) | 25 (47.2) | 10 (38.5) |

Supplementary Table 5. Acknowledgements

The authors are deeply grateful to all study sites, investigators, and supporting staff whose dedication and collaboration made this research possible.

| **Site** | **Investigators** |
| --- | --- |
| University of Virginia Cancer Center, Charlottesville, VA, USA | Elizabeth M. Gaughan |
| Seoul National University Hospital, Seoul, South Korea | Miso Kim, Tae Min Kim, Bhumsuk Keam |
| Peter MacCallum Cancer Centre, Melbourne, VIC, Australia^1^ | Aparna Rao |
| University of California San Francisco, San Francisco VA Health Care System, San Francisco, CA, USA | Maria Wei, Alexandra So, Xiaochen Zhong |
| Addenbrooke’s Hospital, Cambridge, UK | Brent O’Carrigan, Catherine Booth |
| Deutsches Zentrum Immuntherapie, Universitätsklinikum Erlangen, CCC Erlangen-EMN, Friedrich-Alexander-Universität Erlangen-Nürnberg, Erlangen, Germany | Carola Berking, Markus V. Heppt |
| Centre Hospitalier Lyon-Sud, Pierre-Benite Cedex, France | Stéphane Dalle |
| Institut Gustave Roussy, Villejuif, France | Caroline Robert |
| Mount Vernon Cancer Centre, Northwood, UK | Salma Alam |
| CHU Grenoble, La Tronche, France | Julie Charles |
| Nuremberg Hospital, Paracelsus Medical University, Nuremberg, Germany | Dirk Debus |
| Gold Coast University Hospital, Southport, QLD, Australia | Marcin Dzienis |
| Weston Park Cancer Centre, University of Sheffield and Sheffield Teaching Hospitals NHS Foundation Trust, Sheffield, UK | Sarah Danson |
| Velindre University NHS Trust, Cardiff, UK | Ricky Frazer, Tessa Davies |
| University Medical Center Hamburg-Eppendorf, Hamburg, Germany, Hamburg, Germany | Christoffer Gebhardt, Glenn Geidel, Inga Hansen, Lina Hildebrandt |
| Heidelberg University, Medical Faculty Heidelberg, Heidelberg, Germany | Jessica C. Hassel |
| Cleveland Clinic, Cleveland, OH, USA | James Isaacs |
| Seoul National University Bundang Hospital, Seoul National University College of Medicine, Seongnam, South Korea | Yu Jung Kim, Koung Jin Suh |
| Centre Eugène Marquis, Rennes Cedex, France | Thierry Lesimple |
| Ambroise Paré hospital, APHP, & EA 4340 "Biomarkers in cancerology and hemato-oncology”, UVSQ, Université Paris-Saclay, Boulogne-Billancourt, France | Phillipe Saiag |
| Massachusetts General Brigham Cancer Institute, Boston, MA, USA | Ryan Sullivan |
| The Melanoma and Skin Cancer Institute, Englewood, CO, USA | Ryan Weight |

^1^ The authors wish to thank Melanoma Research Victoria (MRV) and acknowledge the MRV sites contributing to this work: the Peter MacCallum Cancer Centre and the Victorian Melanoma Service, Alfred Health.

## Supplementary Figures

Supplementary Figure 1. Treatments by setting and country in the aggregate cohort

Supplementary Figure 2. Anti-PD-1 Cohort: Time to permanent discontinuation of the index LOT in A) the adjuvant setting and B) the advanced setting.
